# Gut microbiota signature as predictors of adverse outcomes after acute ischemic stroke in patients with hyperlipidemia

**DOI:** 10.3389/fcimb.2022.1073113

**Published:** 2022-11-24

**Authors:** Jiaxin Chen, Beibei Chi, Jiaying Ma, Junmei Zhang, Qilu Gu, Huijia Xie, Yu Kong, Shanshan Yao, Jiaming Liu, Jing Sun, Songfang Chen

**Affiliations:** ^1^ Department of Geriatrics, the Second Affiliated Hospital and Yuying Children’s Hospital of Wenzhou Medical University, Wenzhou, Zhejiang, China; ^2^ Department of Preventive Medicine, School of Public Health and Management, Wenzhou Medical University, Wenzhou, Zhejiang, China; ^3^ Department of Neurology, the Second Affiliated Hospital and Yuying Children’s Hospital of Wenzhou Medical University, Wenzhou, Zhejiang, China

**Keywords:** acute ischemic stroke, hyperlipidemia, post-stroke poor outcome, gut microbiota, ROC curve

## Abstract

**Introduction:**

The alterations of gut microbiota have been associated with multiple diseases. However, the relationship between gut microbiota and adverse outcomes of hyperlipidemic stroke patients remains unclear. Here we determined the gut microbial signature to predict the poor outcome of acute ischemic stroke (AIS) with hyperlipidemia (POAH).

**Methods:**

Fecal samples from hyperlipidemic stroke patients were collected, which further analyzed by 16s rRNA gene sequencing. The diversity, community composition and differential gut microbiota were evaluated. The adverse outcomes were determined by modified Rankin Scale (mRS) scores at 3 months after admission. The diagnostic performance of microbial characteristics in predicting adverse outcomes was assessed by receiver operating characteristic (ROC) curves.

**Results:**

Our results showed that the composition and structure of gut microbiota between POAH patients and good outcome of AIS with hyperlipidemia (GOAH) patients were different. The characteristic gut microbiota of POAH patients was that the relative abundance of *Enterococcaceae* and *Enterococcus* were increased, while the relative abundance of *Lachnospiraceae*, *Faecalibacterium*, *Rothia* and *Butyricicoccus* were decreased. Moreover, the characteristic gut microbiota were correlated with many clinical parameters, such as National Institutes of Health Stroke Scale (NIHSS) score, mean arterial pressure, and history of cerebrovascular disease. Moreover, the ROC models based on the characteristic microbiota or the combination of characteristic microbiota with independent risk factors could distinguish POAH patients and GOAH patients (area under curve is 0.694 and 0.971 respectively).

**Conclusions:**

These findings revealed the microbial characteristics of POAH, which highlighted the predictive capability of characteristic microbiota in POAH patients.

## Introduction

Acute ischemic stroke (AIS) was a leading cause of death and chronic disability worldwide. Stroke survivors frequently had various complications, such as cognitive impairment and physical disability, which had a great impact on the quality of life (Duncan et al., 2021; Paul and Candelario-Jalil, 2021). Recent studies have shown that some risk factors including age, smoking and hyperlipidemia could affect the functional outcome after stroke (Meschia and Brott, 2018; Diener and Hankey, 2020). Hyperlipidemia could result in the neuroinflammation of brain and aggravated ischemic brain injury (Kim et al., 2014), and half of stroke patients were found to have hyperlipidemia (Rother et al., 2008). Hyperlipidemic stroke patients might suffer from functional deterioration after AIS. Kim et al. reported that the elevated plasma cholesterol levels were positively correlated with stroke severity in the hyperlipidemic mice (Kim et al., 2020). Elevated low-density lipoprotein cholesterol (LDL-C) was independently associated with severe stroke in patients with chronic kidney disease (Zhang et al., 2021). Currently, early detection of poor outcome of AIS with hyperlipidemia (POAH) was often challenging. Therefore, it is very urgent to find early biomarkers to evaluate the prognosis of hyperlipidemic stroke patients.

Recent studies have emphasized that the characteristic gut microbiota (GM) are associated with AIS. It was reported that stroke patients showed significant dysbiosis of bacteria with enriched short-chain fatty acids (SCFAs) (Li et al., 2019). Our previous studies showed that Proteobacteria was highly increased in the post-stroke cognitive impairment patients compared with the post-stroke noncognitive impairment patients (Ling et al., 2020). More and more evidence showed that GM have important influences on the occurrence, development and severity of stroke. Zhu et al. reported that GM directly impact cerebral infarct size and adverse outcomes following stroke through GM-derived metabolite trimethylamine-N-oxide (Zhu et al., 2021). GM have been increasingly recognized as vital determinants involved in the development of stroke and hyperlipidemia (Ling et al., 2022). The patients with hyperlipidemia showed abnormal GM composition (Gargari et al., 2018), which would aggravate dyslipidemia (Deng et al., 2019; Gu et al., 2020), while regulating GM could alleviate the abnormality of serum lipid in animal models (Yan et al., 2022). These findings demonstrated that GM might be an important regulator of the prognosis of hyperlipidemic stroke patients.

Recent evidences demonstrate that GM could be regarded as a diagnosis biomarker for many diseases. Our previous studies showed that patients with post-stroke comorbid cognitive impairment and depression exhibited an increased abundance of Proteobacteria, and a decreased abundance of several SCFAs-producing bacteria (Ling et al., 2020). It was reported that the abundance of *Alcaligenaceae* and *Acinetobacter* could remarkably distinguish autism spectrum disorders from the healthy group (Li et al., 2019). GM could distinguish stroke patients from healthy controls and the level of SCFAs appeared to effectively predict the severity and prognosis of stroke to some extent (Sun et al., 2021; Tan et al., 2021). The increased relative abundance of *Finegoldia magna, Bifidobacterium dentium*, and *Clostridium clostridioforme* could be used as a predictor of aging (Chen et al., 2022). Although the diagnostic application of GM has been well studied, the characteristic microbiota in POAH patients remains unclear.

Therefore, the present study was performed to investigate the characteristic GM of POAH patients. We further confirmed the correlation between characteristic GM and clinical parameters, as well as determined the gut microbial signature to predict POAH.

## Materials and methods

### Study patients

This study was conducted in the Department of Neurology of the Second Affiliated Hospital of Wenzhou Medical University, from September 2020 to July 2021. Inclusion criteria: patients diagnosed with AIS; admission within 72 hours after stroke onset; previously diagnosed with hyperlipidemia or triglyceride (TG) > 2.28 mmol/L or total cholesterol (TC) > 6.2 mmol/L or high-density lipoprotein (HDL) < 0.91 mmol/L or low-density lipoprotein (LDL) > 3.4 mmol/L. Exclusion criteria: application of antibiotics or probiotics within three months, restriction of diet, concurrent pregnancy, schizophrenia, bipolar disorder, or other serious life-threatening illnesses (heart failure, respiratory failure, or severe renal dysfunction). The modified Rankin Scale (mRS) was applied to assess the post-stroke functional outcome of each patient in a 90-day follow-up after the stroke onset. The included AIS with hyperlipidemia were divided into the good functional outcome group (mRS score < 3) and the poor functional outcome group (mRS score ≥ 3).

### Clinical data collection

All hyperlipidemic stroke patients were collected basic information at enrollment, including sex, age, years of education, history of smoking and drinking, presence of hypertension and diabetes, and history of cerebrovascular disease. Hypertension was considered as blood pressure ≥ 140/90 mmHg. Diabetes was defined as fasting blood glucose ≥ 7.0 mmol/L or 2 h blood glucose ≥ 11.1 mmol/L in an oral glucose tolerance test. The blood samples were extracted on an empty stomach after fasting overnight and centrifuged at 1300xg for 10 minutes. The biochemical indicators analyzed included TG, TC, HDL, LDL, creatinine, vitamin B12, folic acid (FOA), uric acid (UA), homocysteine (Hcy), C-reactive protein (CRP), hypersensitive C-reactive protein (hs-CRP), fasting blood glucose (FPG), glycosylated hemoglobin, thyrotropin, free triiodothyronine (FT3), free tetraiodothyronine (FT4), mean arterial pressure (MAP), D-dimer, alanine transaminase (ALT), aspartate transaminase (AST) and troponin. Moreover, computed tomography (CT) and magnetic resonance imaging (MRI) were used to identify new lesions of patient. Stroke severity was evaluated based on the National Institutes of Health Stroke Scale (NIHSS) by professional physicians within 24 hours of admission. Sleep condition was also quantified through Pittsburgh Sleep Quality Index (PSQI) during hospitalization.

### GM analysis

Fresh stool samples (200 mg) were obtained, and fed into a labeled 2 ml sterile centrifuge tube and quickly stored in a -80°C freezer. The bacterial DNA was isolated by E.Z.N.A. ^®^ Manual of soil Kit (Omega Bio-tek, Norcross, GA, U.S.), and the concentration and purity of which were detected with NanoDrop2000 UV-vis spectrophotometer (Thermo Scientific, Wilmington, USA). The hypervariable regions of the 16s rRNA gene were amplified using PCR with primers 338F: ACTCCTACGGGAGGCAGCAG and 806R:GGACTACHVGGGTWTCTAAT. Next, PCR products were recycled by 2% agarose gel, and paired-end sequenced (2 × 300) on an Illumina MiSeq platform (Illumina, San Diego,USA). Alpha diversity was analyzed through Shannon and ACE. Principal coordinates analysis (PCoA) on the Bray-Curtis dissimilarity index was used for beta diversity analysis. The intestinal typing analysis was performed at the genus level by clustering samples with similar dominant microbiota structures into a class. Moreover, we identified the significant differences in relative abundance at levels of phylum, class, order, family, genus, and species by Wilcoxon rank sum tests based on the obtained community abundance data. Linear discriminant analysis (LDA) effect size (LEfSe) was applied to find significantly enriched taxa and their influence between the two groups using nonparametric Kruskal Wallis (KW) sum rank test, with thresholds of LDA score > 2.

### Statistical analysis

Statistical analysis was carried out by SPSS V.22.0 (SPSS, Chicago, USA). Chi-square test and multivariate logistic analysis were used to analyze the categorical variable data. Odds ratio (OR) and 95% confidence interval (95% CI) were figured out. The values of continuous variables were represented as median with quartile or mean with standard deviation (SD) based on the fact whether they were normally distributed, and compared by rank sum test or t-test respectively. The *P* value < 0.05 was considered to be of significance.

## Results

### Baseline characteristics of the recruited patients

According to the follow-up mRS results, 231 hyperlipidemic stroke patients were divided into two groups: 58 POAH patients and 173 good outcomes of AIS with hyperlipidemia (GOAH) patients. As showed in **Table 1**, POAH patients had significantly elevated levels of age, history of cerebrovascular disease, CRP, hs-CRP, NIHSS score, D-dimer and mRS score compared with GOAH patients. Additionally, a reduction of FT3, MAP and ALT was observed in POAH versus GOAH. There were no statistical differences in demographic data, including gender, educational level, history of smoking and drinking, diabetes, hypertension, and hyperlipemia between the two groups. As shown in **Table 2**, the multivariate logistic regression analysis of demographic and clinical parameters with significant differences described above. The results indicated that a history of cerebrovascular disease (OR = 4.669, p = 0.008), increased NIHSS score (OR = 1.524, *P* < 0.001), and decreased MAP (OR = 0.842, *P* < 0.001) were the independent risk factors of POAH.

**Table 1:** Baseline characteristics of the recruited patients.

Parameter	GOAH group	POAH group	*P*
	(n=173)	(n=58)	
Male (%)	117 (67.6)	35 (60.3)	0.473
Age (years old)	64.03 ± 12.28	70.91 ± 10.77	<0.001
Educational level			0.175
Illiteracy	32	16	
Primary school	64	20	
Junior high school	53	17	
High school and above	24	5	
Smoking	67 (38.7)	17 (29.3)	0.198
Drinking	57 (32.9)	13 (22.4)	0.132
Hypertension	130 (75.1)	46 (79.3)	0.520
Diabetes	67 (38.7)	29 (50.0)	0.133
Hyperlipemia	126 (72.8)	44 (75.9)	0.651
Cerebrovascular disease	28 (16.2)	24 (41.4)	<0.001
Creatinine (μmol/L)	66.30 (55.45-78.65)	62.25 (52.03-76.33)	0.099
Vitamin B12 (pg/mL)	339 (224–436)	350.5 (238-533.25);	0.286
Folic acid (ng/mL)	8.82 (6.77-11.40)	8.65 (5.88-10.53);	0.397
Uric acid (μmol/L)	323.0 (261.0-386.5)	313.5 (241.3-391.5)	0.301
Hcy (μmol/L)	11.40 (9.60-13.99)	11.30 (8.88-14.00)	0.500
CRP (mg/L)	3.30 (2.98-6.05);	4.40 (3.13-13.35)	0.005
Hs-CRP (mg/L)	1.70 (0.94-4.83);	4.39 (1.80-10.00)	<0.001
Triglycerides (mmol/L)	1.68 (1.27-2.23)	1.56 (1.04-2.24)	0.200
Total cholesterol (mmol/L)	4.52 (3.57-5.27)	4.78 (3.61-5.69)	0.263
LDL (mmol/L)	2.94 (2.15-3.67)	3.32 (2.32-3.81)	0.203
HDL (mmol/L))	0.87 (0.77-1.04)	0.91 (0.77-1.19)	0.364
Fasting plasma glucose (mmol/L)	5.49 (4.84-6.64)	5.75 (4.91-7.14)	0.317
Glycosylated hemoglobin (%)	6.11 (5.60-7.02)	6.03 (5.63-7.83)	0.342
Thyrotropin (μIU)	1.91 (1.19-3.02)	1.99 (1.12-2.77)	0.793
FT3 (pg/mL)	2.98 (2.76-3.25)	2.76 (2.53-2.91)	<0.001
FT4 (ng/dL)	1.17 (1.05-1.30)	1.14 (1.07-1.24)	0.959
NIHSS score	2.0 (1.0-3.5)	4.50 (2.75-9.00)	<0.001
PSQI score	5.0 (3.0-8.0)	6.14 (4.0-6.14);	0.105
MAP (mmHg)	139.61 ± 17.03	109.24 ± 11.27	<0.001
D-dimer (mg/L)	0.35 (0.26-0.52)	0.48 (0.33-0.75)	0.003
ALT (μ/L)	17 (13–26)	15.00 (11-21.25)	0.028
AST (μ/l)	18 (15-23)	18.00 (13.75-24)	0.669
Troponin (μmol/L)	0.012 (0.012-0.013)	0.012 (0.012-0.019)	0.396
mRS score	1 (0-2)	3 (3-4)	<0.001

POAH, poor outcomes AIS with hyperlipidemia; GOAH, good outcomes AIS with hyperlipidemia; Hcy, homocysteine; CRP, C-reactive protein; Hs-CRP, hypersensitive C-reactive protein; LDL, low-density lipoprotein; HDL, high-density lipoprotein; FT3, free triiodothyronine; FT4, free thyroid hormone; NIHSS, National Institutes of Health Stroke Scale; PSQI, Pittsburgh Sleep Quality Index; MAP, mean arterial pressure; ALT, alanine transaminase; AST, aspartate transaminase; mRS, modified Rankin scale.

**Table 2:** Multivariate logistic regression analysis.

Parameter	B (SE)	*P*-value	OR	95%CI
Age	0.02 (0.024)	0.271	1.026	0.980-1.075
Cerebrovascular disease	1.541 (0.022)	0.008	4.669	1.486-14.672
Hs-CRP	0.08 (0.085)	0.325	1.087	0.921-1.284
CRP	-0.01 (0.015)	0.245	0.983	0.955-1.012
FT3	0.343 (0.733)	0.639	1.410	0.335-5.925
NIHSS score	0.42 (0.110)	<0.001	1.524	1.228-1.892
MAP	-0.17 (0.029)	<0.001	0.842	0.795-0.892
ALT	-0.01 (0.014)	0.348	0.987	0.961-1.014

Hs-CRP, hypersensitive C-reactive protein; CRP, C-reactive protein; FT3, free triiodothyronine; NIHSS, National Institutes of Health Stroke Scale; MAP, mean arterial pressure; ALT, alanine transaminase; OR, odds ratio; 95%CI, 95% confidence interval.

### Analysis of GM diversity of POAH

**Figure 1:**
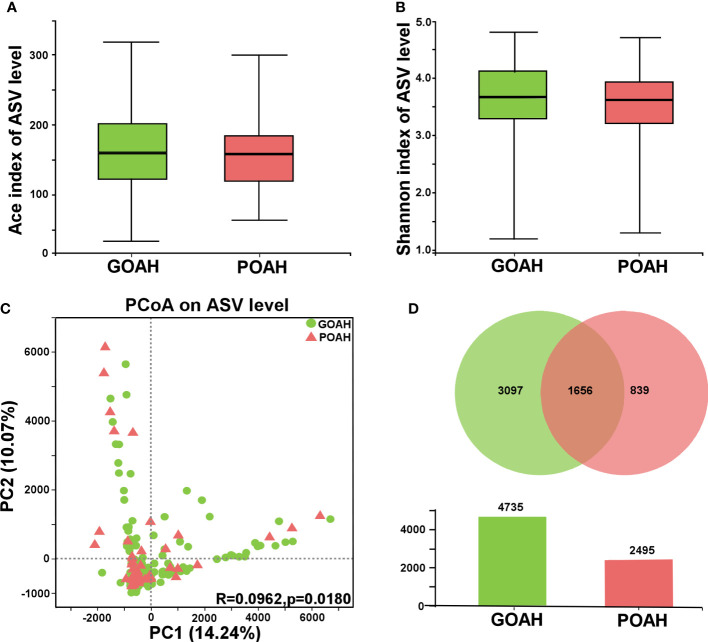
Analysis of gut microbiota diversity of POAH. **(A, B)** Alpha diversity indices, including Ace index and Shannon index. **(C)** Principal coordinate analysis (PCoA) diagram of gut microbiota based on the distance matrix of Bray Curtis (PC1 = 14.24%, PC2 = 10.07%). **(D)** Venn and Bar diagrams showed the number of unique ASVs in GOAH group (green) and POAH group (light red) and their shared ASVs (dark red).

Alpha diversity was evaluated by the Ace index (p = 0.4627, **Figure 1A**) and Shannon index (p = 0.1218, **Figure 1B**), exhibited no significant difference between the two groups. β diversity of the POAH differed from the GOAH according to the PCoA scatterplot (p = 0.018, **Figure 1C**). The Venn and the Bar diagrams exhibited the number of ASVs in the two groups, with 1656 shared ASVs (**Figure 1D**). The number of unique ASVs in GOAH group was 3097, which was higher than the number 839 in POAH.

### Analysis of microbial composition of POAH

**Figure 2:**
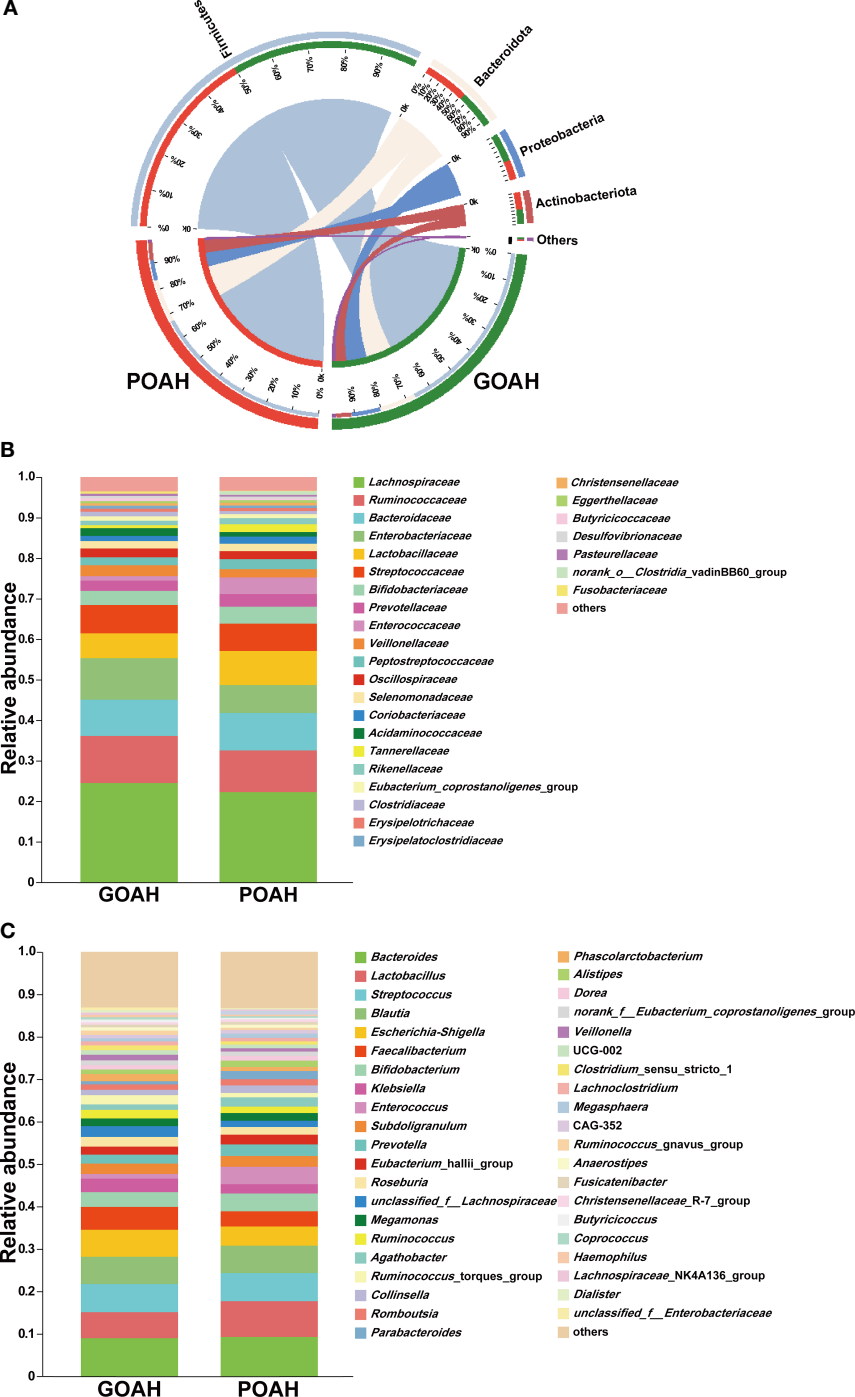
Analysis of microbial composition of POAH. **(A)** Microbial composition at the phylum level. The red bands represent the proportion of phyla in the POAH group. The green bands represent the proportion of phyla in the GOAH group. **(B)** Microbial composition at the family level. **(C)** Microbial composition at the genus level.

As shown in **Figure 2**, the microbial population of phylum level was mainly composed of Firmicutes, Bacteroidota, Proteobacteria and Actinobacteriota (**Figure 2A**). The proportion of Proteobacteria was 55% in the GOAH group. At the family level, the bacterial composition was primarily dominated by *Lachnospiraceae*, *Ruminococcaceae*, *Bacteroidaceae*, *Enterobacteriaceae, Lactobacillaceae*, *Streptococcaceae*, *Bifidobacteriaceae, Preotellaceae*, *Enterococcaceae*, *Veillonellaceae* (**Figure 2B**). And the abundant of the top ten genera that occupied the most of the total microbiota were *Bacteroides*, *Lactobacillus*, *Streptococcus*, *Blautia*, *Escherichia-Shigella*, *Faecalibacterium*, *Bifidobacterium, Klebsiella*, *Enterococcus*, *Subdoligranulum* (**Figure 2C**).

### Analysis of characteristic microbiota of POAH

**Figure 3:**
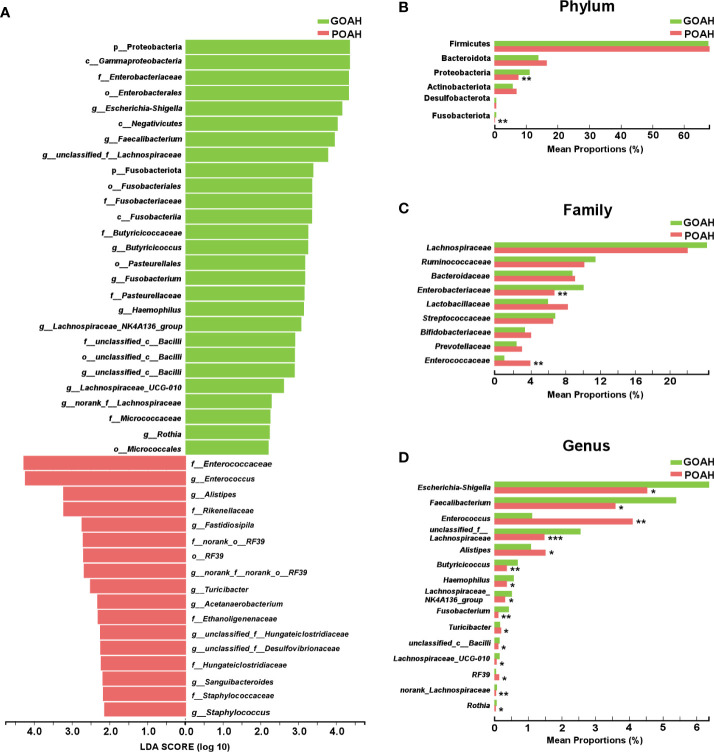
Analysis of characteristic microbiota of POAH. **(A)** Distribution diagram of linear discriminant analysis (LDA) scores of gut microbiota. (LDA > 2). **(B–D)** The extended error bar plot showed significant differences in gut microbial abundance at the level of phylum, family and genus. ^*^: *P* < 0.05, ^**^: *P*< 0.01, ^***^: *P* < 0.001.

As shown in **Figure 3A**, significant bacterial differences in the taxa of the two groups, mainly including *Enterococcaceae*, *Enterococcus*, *Alistipes*, *Rikenellaceae*, *RF_39*, *Turicibacter*, *Acetanaerobacterium*, *Ethanoligenenaceae*, *Hungateiclostridiaceae*, *Sanguibacteroides*, *Staphylococcaceae*, *Staphylococcus* in POAH, and Proteobacteria, *Gammaproteobacteria*, *Enterobacteriaceae*, *Enterobacterales*, *Escherichia-Shigella*, *Negativicutes*, *Faecalibacterium*, *unclassified_f_Lachnospiraceae*, Fusobacteriota, *Fusobacteriales*, *Fusobacteriaceae*, *Fusobacteriia*, *Butyricicoccaceae*, *Butyricicoccus*, *Pasteurellaceae*, *Fusobacterium*, *Pasteurellaceae*, *Haemophilus*, *Lachnospiraceae_NK4A136*, *Bacilli*, *Lachnospiraceae_UCG-010*, *norank_f_Lachnospiraceae*, *Micrococcaceae*, *Rothia* and *Micrococcales* in GOAH. As shown in **Figures 3B–D**, the relative abundance of *Enterococcaceae*, *Alistipes, Turicibacter*, *Enterococcus* and RF39 were higher in the POAH group than GOAH group, while the relative abundance of Proteobacteria, Fusobacteriota, *Enterobacteriaceae*, *Escherichia-Shigella*, *Faecalibacterium*, *Lachnospiraceae*, *Butyricicoccus*, *Haemophilus*, *Lachnospiraceae_NK4A136_group*, *Fusobacterium*, *Bacilli*, *Lachnospiraceae_UCG-010* and *Rothia* were lower in POAH group than GOAH group.

### Analysis of correlation between GM and mRS scores

**Figure 4:**
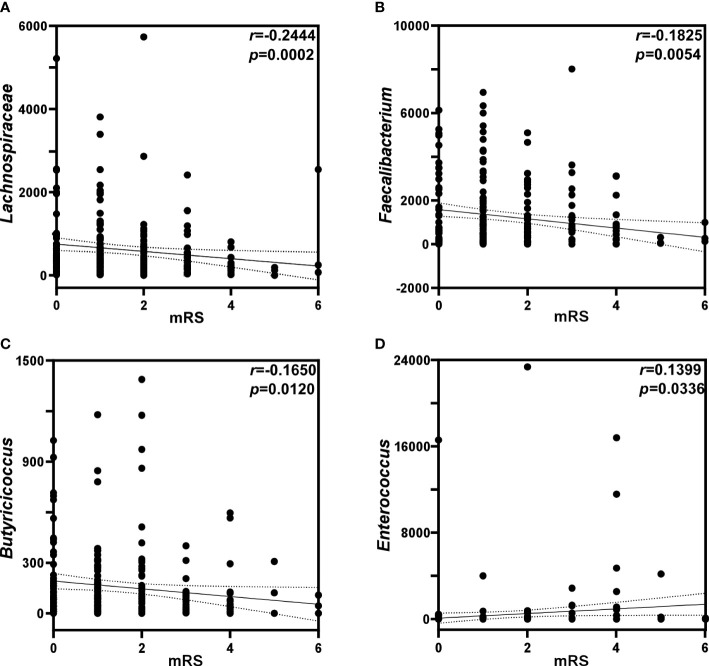
Analysis of correlation between gut microbiota and mRS scores. Correlations of mRS scores with the relative abundance of **(A)** *Lachnospiraceae*, **(B)** *Faecalbiacteruim*, **(C)** *Butyricicoccus*, and **(D)** *Enterococcus*. *p*: probability; *r*: Spearman’s rank correlation.

**Figure 5:**
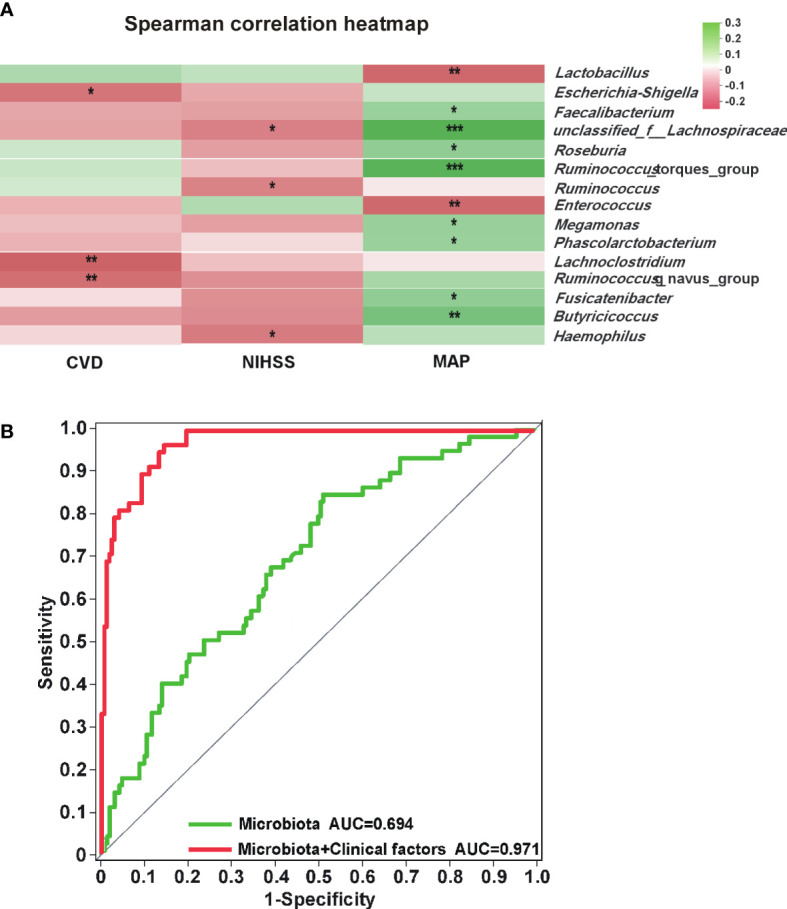
Analysis of correlation between gut microbiota and independent risk factors. **(A)** Heatmap of gut microbiota and independent risk factors for POAH. The colors of grids represent the correlation value of Spearman’s rank correlation analysis. Green grids mean positive correlations, and red grids mean negative correlations. The deeper green or red indicates higher correlation values. ^*^: *P* < 0.05; ^**^: *P* < 0.01; ^***^: *P* < 0.001. **(B)** The green ROC model indicated the predicted value of the composite of five characteristic gut microbiota. The red ROC model was built to evaluate the accuracy based on the complex of five characteristic gut microbiota and three independent risk factors.

As shown in **Figure 4**, *Lachnospiraceae* (*P* < 0.01), *Faecalibacterium* (*P* < 0.01) and *Butyricicoccus* (*P* < 0.05) were negatively correlated with the mRS score, while *Enterococcus* was positively correlated with the mRS score (*P* < 0.05). Spearman correlation heatmap (**Figure 5A**) indicated significant associations between the three independent risk factors and GM. A history of cerebrovascular disease (CVD) was negatively correlated with *Escherichia-Shigella*, *Lachnoclostridium* and *Ruminococcus_gnavus_group*. An elevated NIHSS score was also associated with a reduction of unclassified_f_*Lachnospiraceae*, *Ruminococcus* and *Haemophilus*. Furthermore, a positive relation was observed in MAP with the abundance of *Faecalibacterium*, *unclassified_f_Lachnospiraceae*, *Roseburia*, *Ruminococcus_torques_group*, *Megamonas*, *Phascolarctobacterium*, *Fusicatenibacter*, and *Butyricicoccus*, and a negative relation with the abundance of *Lactobacillus*, *Enterococcus*.

### Analysis of correlation between GM and independent risk factors

We screened out the five genera as biomarkers according to the LDA value, including *unclassified_f_Lachnospiraceae*, *Enterococcus*, *Faecalibacterium*, *Lachnospiraceae_UCG-010*, and *norank_f_Lachnospiraceae*, achieving AUC values of 0.694 (**Figure 5B**, *P* < 0.001, 95% CI 0.618 - 0.770). Moreover, the predictive model combined with the five genera and the three independent risk factors could also distinguish POAH from GOAH (**Figure 5B**, *P* < 0.001, AUC = 0.971, 95% CI 0.952 - 0.989).

## Discussion

This study revealed that GM feature of POAH was that the abundance of *Enterococcus* increased while the abundance of bacteria producing SCFAs decreased, which was closely related to independent risk factors, such as cerebrovascular history, NIHSS score, and MAP. Moreover, the characteristic microbiota and microbiota plus with the three independent risk factors could establish a distinction for predicting POAH. These results indicated that GM might provide novel microbial biomarkers for predicting POAH.

Our results showed that the composition and structure of microbiota were different between POAH and GOAH. Previous studies revealed that gut microbial communities in the group with adverse prognosis after stroke were distinct from those in the group with good prognosis, accompanied by an increase in the abundance of Bacteroidota, and Actinobacteriota, and the decreased abundance of Proteobacteria and the Bacteroidetes to Firmicutes ratio (B/F) (Benakis et al., 2016; Singh et al., 2016; Shimizu et al., 2019; Guo et al., 2021). The diversity of GM was affected by many factors, such as lipid homeostasis (Schoeler and Caesar, 2019). The decreased B/F induced dyslipidemia, leading to more severe outcomes, such as obesity and liver steatosis (Hussain et al., 2020). Our results showed that the abundance of *Enterococcus* in POAH was enriched, and positively related to the mRS score, indicating that the abundance of *Enterococcus* might be related to the risk of POAH. It was reported that *Enterococcus* was an opportunistic pathogen in the gastrointestinal tract, and the risen level of *Enterococcus* was relevant to many neurological and metabolic diseases, such as Parkinson’s disease, Alzheimer’s disease and diabetes (Underly et al., 2015; Li et al., 2017). *Enterococcus* appeared in subjects of the adverse outcome group, manifested as the post-stroke cognitive impairment (PSCI) and post-stroke affective disorder (Huang et al., 2021), which was consistent with our studies. *Enterococcus* could induce the secretion of proinflammatory cytokines, such as IL-6 (García-Solache and Rice, 2019), and further contribute to systemic inflammation (Stanley et al., 2016; Chen et al., 2019), which led to POAH (Suda et al., 2018). Evidence showed that *Enterococcus faecalis* disturbed the lipid metabolism (Huang et al., 2018; Zhu et al., 2021). Hu X et al. revealed that a higher abundance of *Enterococcus* had a closely related to poor prognosis of hypertriglyceridemia-related acute pancreatitis leading to poor prognosis in hypertriglyceridemia patients (Hu et al., 2021), suggesting that *Enterococcus* might be involved in the prognosis of hyperlipidemic stroke patients.

In this study, there was a significantly lower relative abundance of SCFAs-producing bacteria in POAH group, such as *Lachnospiraceae*, *Faecalibacterium*, *Rothia* and *Butyricicoccus*. Moreover, *Lachnospiraceae*, *Faecalibacterium*, and *Butyricicoccus* were associated with lower mRS score. *Lachnospiraceae*, a primary producer of butyrate, was related to the functional prognosis of diseases (Sorbara et al., 2020). Many studies showed that the abundance of *Lachnospiraceae* was significantly decreased in stroke patients and animal models (Zeng et al., 2019; Lin et al., 2021). The abundance of *Lachnospiraceae* in patients with post stroke cognitive impairment (Ling et al., 2020) and patients with nervous neurocritical illness (Xu et al., 2019) was less. In addition, lower blood lipid could increase the abundance of *Lachnospiraceae* and levels of SCFAs in hyperlipidemia model animals (Gui et al., 2019; Liu et al., 2021). In addition, our results showed that the relative abundance of *Faecalibacterium* in POAH group was significantly lower. *Faecalibacterium* is a butyrate-producing bacteria, belonging to *Lachnospiraceae* family. Previous studies showed that the relative abundance of *Faecalibacterium* had a lower relative abundance in patients with stroke (Silveira-Nunes et al., 2020), transient ischemic attack (Yin et al., 2015) and PSCI (Huang et al., 2021) was lower. Lee et al. reported that *Faecalibacterium prausnitzii* ameliorated post-stroke neurological deficits and elevated concentrations of intestinal SCFAs in aged mice with stroke (Lee et al., 2020). *Faecalibacterium prausnitzii* was decreased in fecal samples of hyperlipidemia adolescents (Gargari et al., 2018), and the abundance of *Faecalibacterium prausnitzii* in patients with mild hypercholesterolemia was significantly negatively correlated with TC and LDL (Xu et al., 2021). Furthermore, *Faecalibacterium* was observably elevated in the hyperlipidemia rats after probiotic intake, which could prevent the progression of hyperlipidemia (Shao et al., 2017). Enriched *Faecalibacterium* could reverse the increase of plasma TG level (Tong et al., 2018), and was positively correlated with plasma concentrations of butyric acid (Khan et al., 2018). *Butyricicoccus*, a butyrate-producing clostridial cluster genus, was related to reduced incidence of hyperlipidemia or hypercholesteremia in patients with colorectal cancer (Han et al., 2019). The abundance of *Butyricicoccus* was negatively correlated with the serum levels of LDL, TG and TC of obese patients, which could be used as a biomarker to predict obesity related lipid metabolism abnormalities (Zeng et al., 2019). Recent multiple studies have shown that SCFAs were closely linked to stroke and dyslipidemia. AIS patients, especially those with more severe stroke (Ling et al., 2020), showed a lack of SCFAs-producing bacteria and decreased levels of fecal SCFAs levels, which led to increased risks of post-stroke infection (Haak et al., 2021) and poor functional outcomes (Tan et al., 2021). Furthermore, the feces of young rats transplantation could effectively increase the concentration of SCFAs, and attenuate the neurological deficit and inflammation after stroke in elderly stroke mice (Lee et al., 2020) and in middle cerebral artery occlusion (MCAO) model rats (Chen et al., 2019). In addition, compared with control, subjects with hypercholesterolemia had a lower level of butyrate, which was negatively correlated with LDL (Granado-Serrano et al., 2019). SCFAs played an important role in reducing the risk of cholesterol and coronary heart disease, and valeric acid was negatively correlated with HDL-C in patients with mild hypercholesterolemia (Xu et al., 2021). These results indicated that decreased SCFAs-producing bacteria, such as *Lachnospiraceae*, *Faecalibacterium*, *Rothia* and *Butyricicoccus* and their metabolites SCFAs might participate in the occurrence of POAH.

Our results showed that the characteristic bacteria in POAH patients were closely related to independent risk factors, such as increased, decreased MAP, and history of cerebrovascular disease. The higher NIHSS scores, the greater the risk of disability, the more serious the neurological impairment, and the larger the area of ischemic lesions (Cucchiara et al., 2019; Cucchiara et al., 2020; Wang et al., 2021). A study showed that stroke patients with a history of hyperlipidemia were associated with a higher NIHSS score on day 7 and were less likely to have neurological improvements (Restrepo et al., 2009). Higher MAP could maintain cerebral perfusion and cerebral blood flow velocity in stroke patients. MAP was found to be positively associated with adverse functional outcomes and recurrence risk in stroke patients. It was reported that there was a positive correlation between MAP and the adverse functional outcome and recurrence risk of stroke patients (Ma et al., 2019). Moreover, GM also had a close connection to the clinical parameters. Our results showed that the decrease of unclassified_f_*Lachnospiraceae* was associated with the increase of NIHSS score, and MAP was positively correlated with the abundance of *Faecalibacterium*, *unclassified_f_Lachnospiraceae*, and *Butyricicoccus*, while negatively correlated with *Enterococcus*. LEfSe was used to support the construction of POAH diagnostic model based on five characteristic genera. In addition, the prediction model based on the combination of five characteristics and three independent risk factors could predict the occurrence of POAH. Therefore, these finding revealed the close relationship between POAH and GM, and the characteristic GM could be used as a biomarker for early prediction of POAH.

However, several limitations of this study should be mentioned. First, this was a small sample observational study conducted in a single center. Meanwhile, we collected fecal sample of patients at a single time point, so we could not observe the dynamic changes of the interaction between GM and these parameters. In addition, the information on the concentration of microbial metabolites, such as SCFAs, was lacked, which was difficult to find out the causal relationship between GM and POAH. Despite these limitations, our study firstly described the Characteristic GM of POAH, which was helpful to understand the role of microbial biomarkers in predicting POAH.

In conclusion, these findings revealed the microbial characteristics of POAH, which were closely related to clinical parameters. The characteristic GM might facilitate the diagnosis of POAH, which highlighted the potential prediction of GM on POAH.

## Data availability statement

The datasets presented in this study can be found in online repositories. The names of the repository/repositories and accession number(s) can be found below: NCBI, PRJNA894329.

## Ethics statement

The protocol of the study was reviewed and approved by The Ethics Committee of the Second Affiliated Hospital of Wenzhou Medical University (LCKY2020-207). The patients/participants provided their written informed consent to participate in this study. Written informed consent was obtained from the individual(s) for the publication of any potentially identifiable images or data included in this article. 

## Author contributions

JL, JS and SC designed the experiments. JC, BC, JM, JZ, QG, HX, YK and SY performed the experiments and conducted the statistical analyses. All authors contributed to the article and approved the submitted version.

## Funding

This work was supported by Clinical Medical Research Project of Zhejiang Medical Association (2022ZYC-D10).

## Conflict of interest

The authors declare that the research was conducted in the absence of any commercial or financial relationships that could be construed as a potential conflict of interest.

## Publisher’s note

All claims expressed in this article are solely those of the authors and do not necessarily represent those of their affiliated organizations, or those of the publisher, the editors and the reviewers. Any product that may be evaluated in this article, or claim that may be made by its manufacturer, is not guaranteed or endorsed by the publisher.
